# Quantitative Postnatal Maturation of the Feline Testis from 6 to 36 Months: A Stereological and DHH Immunomorphological Analysis

**DOI:** 10.3390/ani16010010

**Published:** 2025-12-19

**Authors:** Paulo Salinas, Daniel Conei, María Angélica Miglino, Erwin Paz

**Affiliations:** 1 Laboratory of Animal & Experimental Morphology, Institute of Biology, Faculty of Sciences, Pontificia Universidad Católica de Valparaíso, Valparaíso 2340025, Chile; 2 Departamento de Procesos Terapéuticos, Facultad de Ciencias de la Salud, Universidad Católica de Temuco, Temuco 4780000, Chile; 3 Department of Animal Anatomy, School of Veterinary Medicine, Universidade de Marília (UNIMAR), Marília 17525-902, Brazil; 4 UWA Institute of Agriculture, School of Biomedical Sciences, The University of Western Australia, Perth, WA 6009, Australia

**Keywords:** testis, stereology, Desert Hedgehog (DHH), feline, Leydig cells, Sertoli cells

## Abstract

Postnatal testicular maturation in domestic cats remains poorly quantified despite its relevance for reproductive biology, veterinary practice, and the management of threatened felid species. This study aimed to characterize age-related changes in testicular structure from six to thirty-six months of age. Testes were collected from clinically healthy cats undergoing routine orchiectomy, and design-based stereology was used to estimate the volumes and densities of the main testicular components, including the seminiferous epithelium, interstitial tissue, Sertoli cells, and Leydig cells. Immunohistochemical detection of Desert Hedgehog, a developmental signaling molecule, was performed to assess interstitial maturation. Testicular volume and the absolute volumes of the seminiferous epithelium, Sertoli cells, and Leydig cells increased significantly with age, while the numerical density of Leydig cells remained stable, indicating hypertrophy rather than proliferation. Desert Hedgehog immunoreactivity declined progressively across age groups, consistent with the transition from immature to mature interstitial cells. Principal component analysis revealed a clear separation between immature and mature testes based on volumetric and density variables. These results demonstrate that feline testicular maturation follows a coordinated pattern of tubular and interstitial growth and provide quantitative reference values useful for reproductive assessment, comparative studies, and conservation programs in domestic and wild felids.

## Simple Summary

Understanding how the testis grows and matures in domestic cats is important for veterinarians, breeders, and conservation programs for wild feline species. Although the development of the male reproductive system in cats has been described in general terms, detailed information about how the internal structures of the testis change with age has been lacking. In this study, we examined testicular tissue from healthy cats between six and thirty-six months old that had been neutered for routine clinical or population-control reasons. By using high-resolution imaging and precise measurements at the microscopic level, we were able to quantify how the cells and tissues inside the testis expand and reorganize as cats grow from early puberty into adulthood. We also measured the presence of a signal produced by supporting cells in the testis, known to guide development, and found that it decreased as the testis reached maturity. Our findings provide a clear picture of the normal postnatal development of the feline testis and offer valuable reference information for clinical assessment, reproductive management, and conservation efforts involving domestic and wild cats.

## 1. Introduction

The study of testicular development in domestic cats (*Felis silvestris catus*) provides important information about male reproductive physiology, with direct implications for comparative reproductive biology, conservation of wild felids, and the refinement of assisted reproductive technologies [1]. Due to their phylogenetic proximity to endangered felids, domestic cats serve as valuable translational models for understanding the morphological dynamics underlying reproductive function in wild species [2]. Although epididymal maturation has recently been described through stereological and morphometric analyses—highlighting postnatal changes in ductal composition and lumen expansion [3]—the structural remodeling of the testis, particularly from puberty to early adulthood, remains poorly characterized in quantitative terms. The testis integrates gametogenic and endocrine functions under hormonal regulation. The seminiferous epithelium, composed mainly of germ cells and Sertoli cells (*Epitheliocytus sustentans*), sustains spermatogenesis through cycles of proliferation and differentiation [4]. In parallel, Leydig cells (*Endocrinocytus interstitialis*) within the interstitial compartment secrete testosterone in response to luteinizing hormone, supporting local spermatogenesis and systemic androgenic effects [5,6]. Alterations in the number, organization, or functionality of these populations, whether by age or pathology, can compromise fertility and emphasizing the need for unbiased quantitative evaluation.

Previous histological descriptions of domestic cats’ testes have largely been qualitative or semiquantitative, often employing subjective terminology such as “atrophy” or “hyperplasia” [7]. While useful for pathology, such approaches fail to detect subtle age-related structural changes or establish objective baselines across early life stages. This limitation is relevant given that male mammals, including felids, undergo continuous testicular remodeling, unlike the abrupt cessation of female reproductive function [8]. In domestic cats, age-associated declines in sperm quality and mating performance have been documented [9,10,11,12], but the anatomical bases of these declines remain underexplored. Stereology offers a rigorous, unbiased framework for quantifying testicular architecture in three dimensions, enabling the estimation of volumetric and surface densities and numerical cell densities from histological sections [13,14]. This approach has revealed critical morphometric correlates of spermatogenic efficiency across species such as rodents, primates, and humans [15,16,17,18]. However, no stereological analyses have addressed the domestic cat testis across postnatal development. Moreover, the role of local morphogens such as Desert Hedgehog (DHH) in the postnatal testis remains poorly characterized in this species. DHH is a secreted morphogen primarily associated with Sertoli cell signaling and plays a critical role in the differentiation and organization of the interstitial compartment during testicular development. As a member of the Hedgehog family, DHH functions as a highly conserved developmental regulator that governs tissue patterning and cellular interactions in both embryonic and adult contexts across vertebrate and invertebrate species [19]. It plays a pivotal role in gonadal development and is implicated in steroidogenesis and spermatogenesis [20]. In murine models, DHH is expressed by Sertoli cells, exerting paracrine effects on peritubular myoid cell differentiation and juxtacrine effects on Leydig cell maturation [21].

To address these gaps, the present study aimed to quantitatively assess postnatal testicular development in domestic cats aged 6 to 36 months, using design-based stereology combined with immunohistochemical detection of DHH. The central research question addressed how the volume and cellular composition of testicular compartments (seminiferous tubules, interstitial tissue, and associated cell populations) change with age in domestic cats and how the spatial distribution of DHH immunoreactivity varies across these stages. We hypothesized that postnatal aging would be associated with progressive structural remodeling of the testis, characterized by increased volumetric density of the seminiferous epithelium, stabilization of Sertoli and Leydig cell populations, and an age-dependent decline in DHH immunoreactivity indicative of interstitial maturation. By integrating quantitative stereology with DHH immunolabeling, this study establishes normative anatomical parameters for the domestic feline testis, contributing to a broader understanding of reproductive maturation and offering a comparative framework for wild felid species and translational applications in reproductive biotechnology

## 2. Materials and Methods

### 2.1. Ethical Statement

All procedures complied with the Chilean Animal Protection Law No. 20.380. No live animals were used for experimental or research-driven interventions in this study. All testicular samples were obtained exclusively from routine orchiectomies performed for clinical or population-control purposes at a local veterinary hospital, entirely independent of the research team. The indication for surgery, anesthesia, perioperative management, and postoperative care were determined solely by licensed veterinarians and for reasons unrelated to scientific research. Consequently, the research team did not handle, manipulate, or make decisions regarding live animals at any stage.

According to international ethical frameworks and national regulations, studies that involve only discarded biological material collected after routine clinical procedures, without influencing animal care or performing research-driven interventions, are exempt from review by an Institutional Animal Care and Use Committee (IACUC) or its Chilean equivalent. For this reason, formal ethics committee approval was not required. All tissues were received post-surgery as anonymized biological waste and were processed strictly under institutional biosafety standards.

### 2.2. Animals and Samples

A total of 25 testes from healthy, sexually intact, hormone therapy-naive domestic cats (*Felis silvestris catus*) of mixed breed were obtained during routine orchiectomy procedures performed at a veterinary hospital in the Valparaíso Region, Chile (latitude 32°02′ S–33°57′ S; longitude 70–72° W). The health status of all animals was verified through pre-surgical clinical evaluations conducted by licensed veterinarians, including assessments of general condition, body temperature, heart and respiratory rates, and the absence of systemic or reproductive pathologies. Medical histories were corroborated by both owner reports and veterinary clinical records to confirm the absence of chronic diseases, infections, or hormonal treatments. Exclusion criteria included evidence of testicular abnormalities such as cryptorchidism, palpable masses, inflammation, or any signs of reproductive dysfunction. Body weight was recorded prior to surgery as an additional health and growth marker (mean ± SD: 3.3 ± 0.66 kg). To avoid seasonal variation in testicular morphology and hormonal status, all specimens were collected during the austral spring (October), when hormonal levels are relatively stable. Animals were stratified into five age groups: 6, 8, 12, 24, and 36 months, with age determined using a combination of owner-reported birthdates, veterinary clinical records (including vaccination schedules), and dental wear patterns when available. While chronological age estimation is relatively robust before 8 months, we acknowledge potential uncertainties in older animals as noted in the literature [22,23]; this consideration was factored into the interpretation of developmental comparisons. Each testis was macroscopically inspected post-collection to ensure the absence of anatomical alterations. Prior to fixation, gross morphometric parameters (length, width, and weight) were recorded. Testes were dissected free of peritesticular fat and connective tissue, then processed within 30 min of surgical excision. Immediate fixation was performed by immersion in 10% buffered formalin (4% formaldehyde) for 24 h at a constant room temperature (22 °C), following standardized fixation protocols established in the epididymal analysis [3], ensuring comparability across both reproductive organs.

### 2.3. Macroscopic Morphometry and Volume Estimation

Testicular length and width were measured with an electronic caliper to calculate the testicular index (T-index = length × width/body weight) [24]. Each testis was weighed individually, and the gonadosomatic index (GSI = testis weight/body weight × 100) was calculated. To determine testicular volume, fresh testes were measured prior to fixation using the immersion method [25], with correction for isotonic saline density (s = 1.0048 g/cm^3^) [26]. Each sample was suspended in a 300 mL graduated cylinder filled with saline, and the displacement volume was recorded with high precision. Also, each individual testis was measured in centimeters (length, width, and height) with the method of external linear measurements.

### 2.4. Histological Processing and Correction of Shrinkage Artifacts

After fixation in 10% buffered formalin (4% formaldehyde) for 24 h at room temperature (22 °C), all testicular tissues were dehydrated in a graded ethanol series, cleared with xylene, and embedded (Paraplast Plus embedding medium; melting point: 54 °C; Sigma-Aldrich Chemical Co., St Louis, MO, USA). Tissue sections of 5 µm thickness were cut using a calibrated motorized rotary microtome (Leica RM2255, Leica Microsystems, Heerbrugg, Switzerland). For each sample, sections were rehydrated by descending alcohol concentrations (100%, 96%, 80%, 70%; each for 15 s), followed by xylene treatment (10 min) and rinsing in distilled water. Only hematoxylin and eosin staining was employed to facilitate digital image analysis and preserve compatibility with stereological point-counting. To correct for tissue shrinkage artifacts, we replicated the calibration methodology [14,27]. The microtome block advance was calibrated by dividing the change in block height by the number of sections cut, yielding the real section thickness. Additionally, local section thickness was systematically assessed at multiple points in each slide using vertical micrometry on the microscope stage. These values were incorporated as weighting factors in stereological estimations to correct for differential shrinkage across specimens. To minimize optical distortion during z-axis measurements, all stereological evaluations employed oil immersion objectives when necessary. Volume and area deformation due to embedding and cutting were assessed by comparing block dimensions before and after embedding and comparing pre-sectioning areas with final tissue areas. These deformation indices informed a correction factor for each sample. Furthermore, to estimate dimensional shrinkage due to processing, we compared external testicular dimensions measured in situ (prior to fixation) with corresponding dimensions obtained after paraffin embedding. This allowed us to calculate a linear shrinkage coefficient that was incorporated into the computation of corrected volume densities. All digital images were acquired using a Leica^®^DM750 microscope with an MC170HD camera (Leica Microsystems, Heerbrugg, Switzerland). Panoramic images were generated using Motic Easy Scan Pro^®^ (Motic Instruments, Richmond, BC, Canada) to ensure uniformity and enable digital annotation for stereological analysis.

### 2.5. Sectioning and Tissue Preparation

**Figure 1:**
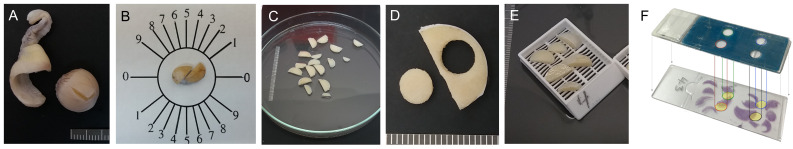
Workflow for obtaining isotropic uniform random sections (IURs) of the domestic cat’s (*Felis silvestris catus*) testis. (**A**) Gross view of the feline testis after dissection and removal of peritesticular connective tissue. (**B**) Application of the Orientator method: a circular template divided into equal sectors was used to generate randomized coordinates for the first cut. (**C**) Serial slabs of approximately 1 mm thickness obtained after systematic slicing of the testis. (**D**) Punching of thin circular sections from randomly selected slabs with a trocar, employed for shrinkage estimation. (**E**) Placement of slabs and circular fragments into histological cassettes prior to routine paraffin embedding. (**F**) Schematic representation of slab embedding and orientation within the paraffin block, illustrating the isotropic distribution of testicular tissue samples used for stereological analysis; yellow-colored areas indicate the stereological sampling sites.

For making isotropic uniform random sections (IURs), the Orientator Method was used [28]. The sectioning was performed according to previously described method [26] with some modifications. Two cuts were made in the testis. The first was performed using a circle divided into equal distances. A random number between 0 and 10 was selected, which indicated the section coordinates, and then the testis was divided into two halves with a single blade. The second cut was performed using a second circle with 10 unequal cosine-weighted divisions. The surface of each half is cut in the direction 0–0. Each half is divided into plates with one blade, placed in the direction of the second cut [28]. The interval is set (~1.0 mm) to generate 6–12 slabs. The newly generated cutting plane was carefully maintained during all histological processing and was used as the histological section plane. For shrinkage estimation one thin and circular section (less than one-tenth of the height of the average-sized particle to prevent overprojection and over estimation) is punched from a randomly sampled slice from a testicular slab by a trocar [26,27]. All the slabs and circular pieces were fixed in 10% buffered formaldehyde and stored for one week, posteriorly embedded in medium. For microscopic evaluation, 5 µm cross sections were obtained in a Leica microtome (RM2255), which were adhered to positively charged slides (Citoglas, Nanjing, China). The sections of testis were processed through a series of alcohols with increasing concentration and cleared in xylene. The paraplast block was cooled and stored at 4 °C until sectioning. Sections of each sample were routinely stained with hematoxylin and eosin and used for immunohistochemistry. All images in this study were taken using a Leica^®^DM750 optical microscope equipped with an Leica^®^MC170HD digital camera (Figure 1).

### 2.6. Morphological and Stereological Analysis

Testicular histological sections were analyzed using an unbiased stereological approach based on systematic uniform random sampling (SURS) [14,29]. In each testis, five histological fields were examined per region (seminiferous tubules and interstitial tissue), totaling 50 fields per testis. All 42 testes obtained in this study were analyzed independently, without selecting a single representative gonad per animal. This strategy ensured the independence of stereological estimations and minimized intra-individual sampling error. The analysis included both morphometric and stereological descriptors. Morphometric parameters, such as seminiferous tubule diameter and epithelial height, were recorded manually using FIJI software (version 2.14.0/1.54f) [30], measuring across circular cross-sections to ensure perpendicularity. Stereological parameters were estimated using the STEPanizer software (version 1.0) [31], employing the M_36_ grid system (36 points, test line = 18 d, test area = 36.36 d^2^) at 10×, 40×, and 100× magnification. The following stereological variables were calculated:

- Volume density (V_V_%) of seminiferous epithelium, tubular lumen, interstitial space, Sertoli cells, and Leydig cells (via point counting).
- Surface density (S_V_) of seminiferous tubules (via intersection counting).
- Numerical density (Q_A_) of Sertoli, Leydig, and germ cells (cells/mm^2^).
- Total volume (V) for each compartment, calculated as V_V_ × corrected testicular volume per sample.

Each testicular sample was analyzed independently by two blinded observers using identical hardware and software after undergoing a calibration and standardization session to harmonize measurement criteria. Prior to analysis, a verification protocol was implemented to assess reproducibility and minimize intraobserver and interobserver bias. Stereological estimations incorporated correction factors derived from shrinkage analysis, ensuring that final values reflected unbiased volumetric proportions. The coefficient of error (CE) was maintained below 5% for all stereological estimators [27]. Histological and anatomical terminology adhered to the *Nomina Histologica Veterinaria* and *Nomina Anatomica Veterinaria* [32,33], ensuring terminological rigor and cross-study comparability.

### 2.7. Immunohistochemistry

Immunohistochemical detection of DHH, a morphogen associated with interstitial differentiation, was performed on paraffin-embedded testicular sections using a polyclonal goat anti-DHH antibody (Santa Cruz Biotechnology, Dallas, TX, USA, N-17). Antigen retrieval was achieved by heat-induced epitope unmasking through steaming in antigen unmasking solution (Vector Laboratories, Newark, CA, USA, H-3301) for 40 min. Endogenous peroxidase activity was quenched by incubation in methanol/H_2_O_2_, and nonspecific binding sites were blocked using phosphate-buffered saline (PBS) supplemented with 3% bovine serum albumin (BSA). Sections were incubated with the primary antibody at a dilution of 1:100, followed by incubation with an HRP-conjugated polymer-based secondary antibody (SuperPicture™, Thermo Fisher Scientific, Waltham, MA USA). Immunoreactivity was visualized using 3,3′-diaminobenzidine (DAB; Vector Laboratories) as chromogen, yielding a brown reaction product. Sections of Sprague–Dawley rat testes were processed in parallel and used as positive controls, whereas negative controls were obtained by omission of the primary antibody. DHH immunoreactivity was consistently localized to the cytoplasm of Leydig cells within the interstitial compartment across all age groups, in agreement with previous immunohistochemical descriptions in mammalian models. No specific or reproducible staining was observed in seminiferous tubular, peritubular, or vascular compartments. Therefore, quantitative analysis was restricted to interstitial Leydig cells to ensure specificity and methodological consistency. Quantitative evaluation was performed to characterize the distribution and relative intensity of DHH immunolabeling, without inferring absolute protein expression levels. For each testis, five non-overlapping microscopic fields were selected using systematic random sampling at 40× magnification. Digital images were acquired under identical illumination, exposure, and acquisition settings using a Leica DM750 microscope equipped with an MC170HD camera. Image analysis was carried out using Image-Pro Plus software (version 9.1; MediaCybernetics, Rockville, MD, USA). Two complementary immunohistochemical parameters were quantified:

(i) The percentage of immunoreactive area (IRA, %), defined as the proportion of the interstitial area occupied by DHH-positive staining, serving as an estimate of the spatial extent of immunolabeled Leydig cells;
(ii) The integrated optical density (IOD) of the DAB signal, reflecting the relative intensity of immunostaining within the immunoreactive area.

Threshold values for DAB detection were established once and applied uniformly across all sections and age groups to minimize operator-dependent variability. For each animal, mean values derived from the five sampled fields were used for statistical analysis. Together, these measurements provided a standardized and reproducible assessment of age-related changes in the immunolocalization pattern and relative immunoreactivity of DHH in Leydig cells, without implying quantitative protein expression.

### 2.8. Statistical Analysis

Descriptive statistics were expressed as mean ± standard deviation (SD) for macroscopic morphometric variables and as median with interquartile range (IQR) for stereological and immunohistochemical parameters. The choice of summary statistics reflected the underlying distribution of the data. Data normality was assessed using the D’Agostino–Pearson omnibus test, and homogeneity of variances was evaluated using Levene’s test. Macroscopic morphometric variables (body weight, testicular weight, testicular indices, and gonadosomatic index) that met assumptions of normality and homoscedasticity were analyzed using one-way analysis of variance (ANOVA), followed by Tukey’s multiple-comparison post hoc test. In contrast, stereological and immunohistochemical variables—including volume density (V_V_), surface density (S_V_), numerical density (Q_A_), integrated optical density (IOD), and percentage of immunoreactive area—did not satisfy normality assumptions and were therefore analyzed using non-parametric Kruskal–Wallis tests followed by Dunn’s post hoc comparisons. To ensure methodological rigor in quantitative histology, coefficients of error (CE) and coefficients of variation (CV) for each stereological estimator were calculated following established recommendations for design-based stereology [34,35], allowing discrimination between biological variability and methodological noise. Correlation analyses were performed using two complementary approaches based on data distribution. Pearson’s correlation coefficient was used to assess linear associations between age and macroscopic morphometric variables that exhibited normal distributions, whereas Spearman’s rank correlation coefficient was applied to associations involving stereological and immunohistochemical parameters that did not meet parametric assumptions. Principal Component Analysis (PCA) was employed as an exploratory multivariate technique to reduce data dimensionality and identify latent patterns associated with postnatal testicular maturation. Prior to PCA, all variables were standardized (z-score transformation) to account for differences in measurement scales. Principal components were retained according to the Kaiser criterion (eigenvalues > 1.0). Two complementary graphical representations were generated: a scores plot to visualize the distribution of individuals and age groups in multivariate space, and a loadings plot to identify the variables contributing most strongly to group separation. All statistical analyses and graphical outputs were performed using GraphPad Prism version 10.0 for macOS (GraphPad Software, San Diego, CA, USA). Statistical significance was set at *p* < 0.05 for all analyses.

## 3. Results

### 3.1. Macroscopic Morphometric Variables

Macroscopic morphometric variables of domestic cats at different ages are summarized in Table 1. Body weight (BW) showed a progressive and statistically significant increase with age, being lowest at 6 months (2.58 ± 0.476 kg) and highest at 36 months (3.91 ± 0.619 kg; one-way ANOVA, *p* = 0.0027), with post hoc analysis indicating significant differences between the 6-month group and the older age groups. Total testicular weight (TW) was also significantly lower at 6 months (36.3 ± 7.71 g) compared with all subsequent ages (ANOVA, *p* = 0.0016), reaching a maximum at 8 months (69.2 ± 12.8 g) and remaining relatively stable thereafter, as indicated by shared post hoc groupings. Similarly, both right and left testicular indices (T-index) exhibited their lowest values at 6 months (right: 39 ± 10.7; left: 35.8 ± 7.67) and significantly higher values in older animals (right T-index: *p* = 0.0026; left T-index: *p* = 0.0023), with Tukey’s post hoc test confirming that the 6-month group differed from the remaining age groups, whereas differences among post-pubertal groups were less pronounced. The gonadosomatic index (GSI) increased markedly with age, from 36 ± 1.18 at 6 months to 130 ± 22.1 at 36 months and showed the strongest age-related effect among all macroscopic parameters (ANOVA, *p* < 0.0001). Post hoc comparisons indicated that GSI values at 6 months were significantly lower than those observed at all later ages, with a progressive increase across age groups. Overall, BW and GSI exhibited the largest relative increases between juvenile and adult stages, whereas TW and T-indices showed an early rise at 8 months followed by a plateau, suggesting stabilization of gross testicular size after the onset of sexual maturity. All statistical inferences are supported by one-way ANOVA followed by Tukey’s post hoc test, with significant pairwise differences indicated by distinct superscript letters in Table 1.

### 3.2. Stereological Parameters of Testicular Compartments

Stereological estimators for testicular structures in domestic cats at different ages are presented in Supplementary File S2 (Table S1). The median reference volume of the testis (Vol_REF_) increased progressively from 0.34 mm^3^ at 6 months to 2.15 mm^3^ at 36 months. The numerical density (Q_A_) of Leydig cells remained approximately constant across most age groups, while their absolute volume (V_ABS_) showed a progressive increase, reaching a maximum median of 0.19 mm^3^/testis at 36 months. Volume fraction (V_V_) of Leydig cells remained stable across groups. The Q_A_ of Sertoli cells ranged from 68.4 to 102.6 cells/mm^2^, while their V_ABS_ increased from 0.08 mm^3^ at 6 months to 0.28 mm^3^ at 36 months. The volume fraction of Sertoli cells (V_V_) showed slight variation, with values between 8.16% and 12.24%. Regarding the spermatogenic epithelium, the Q_A_ ranged between 1618 and 2166 cells/mm^2^ across age groups. The absolute volume (V_ABS_) of this compartment increased with age, from 0.23 mm^3^ at 6 months to 0.76 mm^3^ at 36 months. The volume fraction (V_V_) of the spermatogenic epithelium ranged between 28.57% and 36.73%. Group variation coefficients (%OCV_GROUP_) and individual estimation error (%OCE_INDIVIDUAL_) for each parameter are also detailed in the table. Statistically significant differences between age groups were found in several parameters (*p* < 0.05), as indicated by matching superscript numbers. A detailed visualization of these stereological dynamics is provided in Supplementary File S1 (Figure S1).

### 3.3. Immunohistochemical Detection of DHH in Leydig Cells

**Figure 2:**
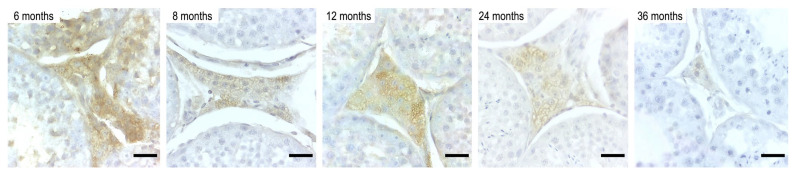
Immunolocalization of Desert Hedgehog (DHH) in the testis of domestic cats (*Felis silvestris catus*) aged 6, 8, 12, 24, and 36 months. DHH immunoreactivity was exclusively detected in the cytoplasm of Leydig cells located within the interstitial compartment. A progressive decrease in both intensity and area of immunostaining was observed with advancing age, with the highest signal at 6 months and minimal reactivity at 36 months. Indirect immunoperoxidase staining using a goat polyclonal anti-DHH primary antibody (N-17, Santa Cruz Biotechnology). Scale bar = 100 µm.

The immunohistochemical localization of Desert Hedgehog (DHH) was observed consistently in the interstitial compartment, specifically within the cytoplasm of Leydig cells in all age groups (Figure 2). Quantitative evaluation of immunoreactivity is presented in Table 2. The median percentage of DHH-immunoreactive area decreased progressively with age, from 18.17% at 6 months to 3.084% at 36 months. Similarly, the IOD of DHH labeling also decreased across age groups, from 0.7442 (±0.02795) at 6 months to 0.2452 (±0.02719) at 36 months. Statistically significant differences were detected among age groups for both parameters (*p* < 0.0001).

### 3.4. Age-Dependent Correlations in Body Weight and Testicular Characteristics

The results presented in Table 3 reveal differential patterns of association between age and various reproductive and somatic parameters in male subjects. A strong and statistically significant positive correlation was observed between age and body weight (*r* = 0.9302, *p =* 0.0219), indicating that body weight increased consistently with advancing age. In contrast, testicular weight and both right and left testicular indices exhibited weak and non-significant correlations with age (*r* ranging from 0.3555 to 0.4344; *p* > 0.46), suggesting limited age-related variation in these reproductive traits. Interestingly, the GSI showed a strong correlation with age (*r* = 0.8453), although it did not reach statistical significance (*p* = 0.0713). This finding may nonetheless indicate a biologically relevant trend that warrants further investigation in studies with greater statistical power.

### 3.5. Principal Component Analysis (PCA)

**Figure 3:**
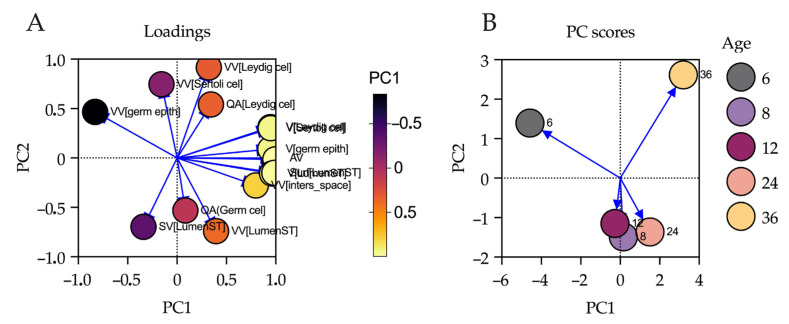
Principal Component Analysis (PCA) applied to stereological and morphometric variables of the testis in domestic cats (*Felis silvestris catus*). (**A**): Loadings Plot. This panel shows the contribution of each variable to the PCA model along PC1 and PC2. Arrows indicate the direction and magnitude of the correlation of each variable with the principal components. The size of the circles reflects the variance explained by PC1 (color) and PC2 (area), respectively. Variables most strongly associated with PC1 (testicular volume, interstitial compartment, and tubular lumen) account for much of the ontogenetic gradient, whereas PC2 is primarily associated with variability in germinal epithelium density and seminiferous lumen proportions. (**B**): Scores Plot. This panel displays the distribution of individuals (*n* = 5 age groups) in the multivariate space defined by the first two principal components. Each point represents an age group and is color-coded according to age (in months). The observed gradient from the 6-month to the 36-month group suggests a clear ontogenetic separation, with progressive clustering of mature groups and greater dispersion among immature individuals.

Principal component analysis was used to explore the multivariate structure of stereological and morphometric variables throughout testicular development. The first two components (PC1 and PC2) jointly explained 78.86% of the total system variance (PC1 = 50.66%; PC2 = 28.20%). The loadings plot (Figure 3A) revealed that the variables with the highest positive loadings on PC1 were the absolute volumes of the Sertoli cell compartment, Leydig cells, germinal epithelium, and tubular lumen, as well as total testicular volume, indicating that this component summarizes the structural expansion associated with gonadal maturation Supplementary File S3 (Table S2). In contrast, PC2 was more strongly influenced by variables related to cell density and the volumetric proportions of the epithelium and seminiferous lumen, suggesting that this axis reflects differences in internal tissue organization rather than absolute size. The scores plot (Figure 3B) illustrated the distribution of individuals by age within the multivariate space defined by PC1 and PC2. A clear ontogenetic gradient was observed from 6 to 36 months, with a progressive separation of age groups. Individuals at 6 months clustered at the negative end of both PC1 and PC2, reflecting an immature profile in terms of both testicular volume and epithelial organization. In contrast, individuals at 36 months were grouped in the opposite quadrant, characterized by high PC1 and low PC2 values, corresponding to greater volumetric expansion and lower relative cell density, consistent with a mature testicular structure. These findings confirm that the stereological variables effectively discriminate between developmental stages of the testis according to age, with the numerical contribution of each variable detailed in Supplementary File S3 (Table S2).

## 4. Discussion

Our results confirm that postnatal aging of the feline testis involves progressive structural remodeling that reaches completion in early adulthood. Macroscopically, the sigmoid increase in testicular volume and gonadosomatic index between 6 and 24 months reflects activation of the hypothalamic–pituitary–gonadal axis and aligns with the pubertal threshold reported in domestic and wild felids [36,37,38,39]. At the tubular level, the marked expansion of the seminiferous epithelium and epithelial height indicates enhanced spermatogenic efficiency, while germ cell numerical density showed only minor fluctuations attributable to the spermatogenic cycle and selective apoptosis [17,40]. The decline in the Sertoli-to-germ cell ratio with age is consistent with Sertoli cell cytoplasmic expansion to sustain higher sperm output [41,42]. Within the interstitial compartment, Leydig cells underwent pronounced hypertrophy without significant changes in numerical density, pointing to functional rather than proliferative adaptation [5,43,44], a mechanism that preserves compartmental balance and ensures stable steroidogenesis during the reproductive lifespan. Concurrently, DHH immunoreactivity decreased steadily from 6 to 36 months. Since DHH secreted by Sertoli cells promotes interstitial precursor differentiation, its attenuation suggests that Leydig cells had reached a mature state with reduced need for morphogenetic signaling [45,46,47,48]. Thus, DHH quantification emerges as a promising biomarker to distinguish immature testes in assisted reproduction and conservation contexts. Together, these findings highlight the domestic cat as a robust model for testicular ontogeny: rapid growth up to two years of age followed by structural stabilization, hypertrophy rather than hyperplasia of Leydig cells, and adjustment of the Sertoli-to-germ cell trophic ratio to sustain spermatogenesis. These stereological references provide a valuable baseline for evaluating reproductive pathologies and guiding biotechnological interventions in threatened felids.

### 4.1. Postnatal Testicular Maturation in the Domestic Cat

The sigmoid increase in testicular volume and GSI between 6 and 24 months confirms that activation of the hypothalamic-pituitary-gonadal axis drives a rapid expansion of both the seminiferous tubules and the interstitial stroma, followed by a structural plateau by 36 months of age. Recent single-cell transcriptomic studies demonstrate that this growth is accompanied by the differential expansion of germ cell populations and the definitive maturation of Sertoli and Leydig cells [49]. A minimum body weight of 2.5 kg and a testicular weight greater than 1 g emerge as predictive thresholds of active spermatogenesis, in accordance with both classical and contemporary andrological findings [1,11]. The GSI showed a strong positive correlation with age (*r* = 0.85), outperforming absolute testicular weight in its discriminative capacity to indicate pubertal onset—an observation previously described in small carnivores and corroborated in the present study [50,51]. The stabilization of both GSI and total testicular volume between 24 and 36 months suggests that the ratio between the tubular and interstitial compartments reaches a state of homeostasis, as documented in comparative analyses of carnivorous and ungulate mammals [41,52,53]. From a histodynamic perspective, the doubling of epithelial volume within the first eight months reflects the transition from an exclusive presence of spermatogonia to the emergence of elongated spermatids, a pattern consistent with findings in wild felids [2,54,55,56,57].

### 4.2. Stereological Interpretation of the Interstitial Compartment

The numerical density of Leydig cells remained stable (~34 cells/mm^2^) throughout postnatal development from 6 to 36 months, whereas their absolute volume increased approximately sixfold (0.03 → 0.19 mm^3^/testis, *p* < 0.0001). This dissociation indicates that interstitial remodeling in the domestic cat is driven primarily by cellular hypertrophy rather than proliferation. Such a pattern reflects a progressive enhancement of Leydig cell functional capacity while preserving numerical stability within the interstitial compartment. This hypertrophic mechanism has been described as a conserved feature of testicular maturation across mammals. Single-cell transcriptomic studies have shown that somatic maturation of Leydig cells is accompanied by increased steroidogenic competence, including upregulation of enzymes such as STAR and CYP11A1, without expansion of the cell population [58,59,60,61]. Consistent with this model, testicular growth in the present study occurred without increases in Leydig cell numerical density, thereby maintaining tubular–interstitial proportionality and preventing excessive tissue expansion [5]. Comparable structural dynamics have been reported in rodents, where luteinizing hormone stimulation induces Leydig cell hypertrophy with minimal changes in numerical density [59,60,61,62], as well as in humans during pubertal maturation. In this context, the age-related reduction in DHH immunoreactivity observed in Leydig cells is interpreted as a morphological correlate of interstitial maturation rather than evidence of active pathway downregulation. Similar patterns have been described in murine models, in which DHH signaling diminishes once Leydig cell differentiation is established [46]. Collectively, these findings indicate that maturation of the interstitial compartment in the domestic cat is characterized by volumetric expansion and functional optimization of Leydig cells, ensuring stable steroidogenic output during early reproductive life.

### 4.3. Dynamics of Sertoli Cells and the Spermatogenic Epithelium

The maturation of the feline seminiferous epithelium exhibits a dissociation between the number and size of Sertoli cells: their numerical density (Q_A_) fluctuates due to tubular reorganization, while their absolute volume increases more than threefold between 6 and 36 months, reflecting cytoplasmic expansion to support an increased germ cell load. Single-cell transcriptomic studies in humans and sheep have demonstrated that, after puberty, Sertoli cells reach a stable “mature” state, characterized by increased expression of androgen receptor (AR), Insulin-Like Growth Factor 1 (IGF1), and lipid transport pathways, without de novo proliferation [59,63,64]. In the domestic cat, this same volumetric, but not numerical, expansion was confirmed through molecular phenotyping (SOX9, GATA1) and 3D stereology [49], supporting the idea that the final Sertoli cell complement is established during the peri-pubertal phase and that the Sertoli-to-germ cell efficiency increases mainly via functional hypertrophy. The absolute volume of the germinal epithelium increased by approximately 3.3-fold, whereas its Q_A_ showed transient oscillations typical of the apoptotic and synchronous waves of the spermatogenic cycle. Recent scRNA-seq atlases describe these oscillations as pulses coordinated by FSH-dependent signaling and Sertoli-germ cell paracrine crosstalk [58]. The Sertoli-to-germ cell ratio gradually decreased to approximately 1:17 in early adulthood, a range considered optimal for efficient spermatogenesis in mammals [6,17,65]. The subsequent stabilization suggests that the feline epithelium reaches a homeostatic equilibrium similar to that observed in rodents and humans, in which the Sertoli cell number remains constant after puberty [17,42,66]. Taken together, our results and the comparative evidence indicate that testicular maturation in the domestic cat relies on (i) progressive hypertrophy of Sertoli cells without numerical renewal, (ii) synchronization of germ cell turnover via controlled apoptotic cycles, and (iii) fine-tuning of the Sertoli-to-germ cell ratio to maximize sperm output while minimizing interindividual variability.

### 4.4. Functional Role of DHH in Interstitial Maturation

DHH immunoreactivity declined in a log-linear manner between 6 and 36 months, both in terms of positive area and IOD, consistent with the transition from progenitor Leydig cells to a mature steroidogenic phenotype. Recent single-cell studies confirm that, in mammals, interstitial cells transiently activate DHH during the commitment phase and downregulate its expression once stable androgen production is established [67,68]. In the domestic cat, this decline is accompanied by cytoplasmic hypertrophy and upregulation of steroidogenic genes (STAR, CYP11A1), further supporting the interpretation of DHH as an inverse marker of functional maturity. Novel in vivo findings in humans reveal that the testicular microenvironment secretes leptin, which stimulates the DHH-PTCH1 pathway in Leydig stem cells; inhibition of DHH abolishes this effect, underscoring its key role as a convergence point between endocrine and paracrine signaling [69]. In murine models, loss-of-function mutations in Nr2f2 impair Leydig cell differentiation without altering DHH signaling, placing this pathway downstream of nuclear regulators of the interstitial lineage [67,70]. Classic mouse studies demonstrated that DHH, secreted by Sertoli cells, induces fetal Leydig cell ontogeny and organizes the peritubular lamina; its expression declines once cells reach full steroidogenic activity [45,46,47,48]. In humans, biallelic mutations in DHH cause gonadal dysgenesis and Leydig cell failure, highlighting the clinical relevance of this pathway [71]. The dynamic observed in *Felis silvestris catus* supports the use of DHH as a biomarker for selecting pubertal donors in felid conservation programs, such as for guiña (*Leopardus guigna*) and margay (*Leopardus wiedii*), where sample mass is limited [72,73]. Thus, standardized monitoring of DHH links comparative anatomy with reproductive biotechnology, enhancing gamete bank management and ex situ population strategies.

### 4.5. Multivariate Analysis: Structural Validation via Principal Component Analysis

PCA enabled a robust integration of the stereological and morphometric data generated in this study, revealing latent patterns of variation associated with postnatal testicular development in *Felis silvestris catus*. The first two principal components accounted for more than 75% of the total variance, indicating high model efficiency in capturing the structural dynamics of gonadal maturation. This multivariate approach has been previously validated in reproductive contexts, allowing for the identification of major axes of variation in studies of spermatogenesis and testicular development [74,75]. Specifically, PC1, which explained 50.66% of the variance, was strongly associated with absolute volume variables such as total testicular volume, Sertoli cell compartment, Leydig cells, germinal epithelium, and tubular lumen. This pattern suggests that PC1 represents an axis of global structural maturation, linked to the progressive increase in the functional mass of the testis. The relevance of these parameters as predictors of testicular maturity has been widely documented in both domestic and wild species [41]. The positive loading of these variables on the same component indicates synchronous structural co-expression, characteristic of physiological gonadal development. PC2, accounting for 28.20% of the variance, was associated with variables reflecting internal tissue organization, such as germ cell density and the volumetric proportions of the seminiferous epithelium and lumen. This suggests that PC2 captures an axis of microstructural reorganization of the seminiferous epithelium, consistent with previous reports describing progressive remodeling of tubular architecture during the transition from puberty to adulthood [17,56,76]. The progressive age-based separation observed in the PCA score plot reinforces the model’s sensitivity in detecting ontogenetic differences, even between closely related age groups such as 8 vs. 12 months, differences that are often undetectable via univariate analyses. The clustering of 6-month-old individuals in the negative quadrant of both components, and their clear separation from 36-month-old individuals, indicates that these two age extremes represent functionally distinct testicular phenotypes: an immature phenotype with low functional mass and high relative density, and a mature phenotype characterized by volumetric expansion and lower cellular density. This pattern reflects a shift from a densely packed cellular architecture to a more expanded and differentiated state, in agreement with stereological models in rats and humans, where spermatogenic efficiency is associated with coordinated changes in volume and tissue organization [77]. Taken together, the PCA quantitatively validates the existence of a morphofunctional gradient associated with age, providing multivariate statistical evidence that testicular maturation in the domestic cat involves a coordinated transformation of volume, density, and proportionality across the main testicular compartments. This approach offers a powerful tool for comparative studies in wild felids, where small sample sizes often necessitate multivariate data integration for robust biological interpretation.

### 4.6. Functional Integration of the Testis and Epididymis: Synchronized Maturation of the Male Reproductive Tract

The present study on the domestic cat testis complements a previous stereological analysis of the epididymis [3], enabling an integrated view of the postnatal maturation of the male reproductive tract. Both organs exhibited a coordinated structural progression between 6 and 36 months, characterized by significant volumetric expansion and reorganization of their functional compartments. In the testis, a sustained increase in the volume of the spermatogenic epithelium and in Sertoli and Leydig cell volumes was observed, concomitant with a progressive reduction in DHH immunoreactivity within the interstitial compartment In parallel, previous stereological analyses of the epididymis in domestic cats have demonstrated a significant increase in luminal diameter and ductal lumen volume during postnatal development [3], suggesting that epididymal and testicular maturation occur in a coordinated manner. These complementary structural modifications suggest that testicular and epididymal maturation occur synchronously, likely responding to shared endocrine signals and the progressive rise in circulating testosterone, as described in other species [6,41]. The expansion of the ductal lumen and the reduction in epithelial density in the epididymis reflect increased secretory activity and enhanced sperm storage capacity, consistent with the progressive activation of the hypothalamic-pituitary-gonadal axis observed in the testis. From a comparative and functional perspective, this integration demonstrates that the domestic cat is a valuable model not only for the isolated study of reproductive organs but also for understanding their functional development as an interdependent system.

### 4.7. Limitations and Future Directions

This cross-sectional study, structured across five critical age groups, provides a detailed description of feline testicular ontogeny; however, it does not capture intraindividual dynamics or the rate of histological change. The sample size (*n* = 5 per group) reflects ethical constraints commonly encountered in stereological studies of medium-sized mammals. Nevertheless, the combination of SURS and the optical fractionator with >100 intersections per case maintained the stereological error below 10%, in accordance with international standards of best practice [13]. The application of multivariate analysis added interpretive power and validated the morphogenetic patterns observed. To avoid seasonal bias, all castrations were performed during the austral spring, the period of maximal gonadal activity in felids. The absence of serum hormonal profiles and sperm parameters was compensated by validated proxies: GSI, stereological estimations, and DHH immunoreactivity, which exhibit strong correlations with testicular function. Nonetheless, future studies should incorporate longitudinal ultrasonographic-hormonal monitoring and spermiograms to refine morphofunctional correlations. While the domestic cat is an accessible and phylogenetically close model, ecological differences among felids necessitate caution when extrapolating these data to endangered species. Despite this, the sampling protocol, design-based stereology, and multivariate analysis employed here constitute a robust and transferable platform for comparative research, conservation, and reproductive biotechnology in wild felids.

## 5. Conclusions

This study quantitatively demonstrated that postnatal maturation of the feline testis between 6 and 36 months is characterized by a progressive and organized remodeling of both tubular and interstitial compartments. Design-based stereology revealed a significant age-related increase in total testicular volume and in the absolute volumes of the seminiferous epithelium, Sertoli cells, and Leydig cells, while the numerical density of somatic cell populations remained largely stable, indicating that testicular growth is driven predominantly by cellular hypertrophy rather than proliferation. In parallel, DHH immunoreactivity exhibited a consistent and marked decline with age, supporting its role as an inverse indicator of interstitial differentiation and maturation. Multivariate analysis further validated these morphofunctional patterns, enabling clear discrimination between immature and mature testes based on integrated volumetric and cellular variables. Collectively, these findings establish normative quantitative parameters for postnatal testicular development in the domestic cat and support its use as a robust comparative model for reproductive biology, with direct relevance for translational research and conservation-oriented studies in wild felids.

## Figures and Tables

**Table 1:** Macroscopic morphometric analysis of the testis of domestic cats (*Felis silvestris catus*) at different postnatal ages (months). Values are expressed as group means ± SD (coefficient of variation in brackets).

	6	8	12	24	36	*p*-Value
BW (kg)	2.58 ^ab^ ± 0.476 [18.5%]	3.02 ± 0.46 [15.2%]	3.3 ± 0.367 [11.1%]	3.58 ^a^ ± 0.39 [10.9%]	3.91 ^b^ ± 0.619 [15.9%]	0.0027
TW (g)	36.3 ^abcd^ ± 7.71 [21.3]	69.2 ^a^ ± 12.8 [18.5]	60.4 ^b^ ± 13.2 [21.9]	66.5 ^c^ ± 10.6 [16.0]	63.5 ^d^ ± 12.7 [19.9]	0.0016
T-index Right	39 ^abcd^ ± 10.7 [27.3]	70 ^a^ ± 14.3 [20.4]	69.9 ^b^ ± 11.9 [17.1]	66.4 ^c^ ± 8.65 [13.0]	65.3 ^d^ ± 13.8 [21.2]	0.0026
T-index Left	35.8 ^abcd^ ± 7.67 [21.4]	67.8 ^a^ ± 12.8 [18.9]	59 ^b^ ± 13.2 [22.4]	64.6 ^c^ ± 10.5 [16.2]	61.1 ^d^ ± 12.5 [20.5]	0.0023
GSI	36 ^abcd^ ± 1.18 [3.29]	90.8 ^ae^ ± 12.6 [13.9]	86.5 ^bf^ ± 9.08 [10.5]	104 ^c^ ± 8.1 [7.78]	130 ^def^ ± 22.1 [17.0]	<0.0001

All parameters correspond to measurements obtained from individual testes. Body weight (BW) refers to the animal body mass. Testicular weight (TW) represents the weight of a single testis. Testicular index (T-index) was calculated separately for the right and left testes as (length × width)/body weight. Gonadosomatic index (GSI) was calculated for each testis as (testis weight/body weight) × 100. Statistical differences among age groups were determined using one-way ANOVA followed by Tukey’s post hoc test (*p* < 0.05). Different superscript letters indicate significant differences between groups.

**Table 2:** Median (SD) values of DHH immunoreactivity in Leydig cells of domestic cats (*Felis silvestris catus*) at different postnatal ages (months), including percentage of immunoreactive area (IRA) and integrated optical density (IOD).

	6 m	8 m	12 m	24 m	36 m	*p*-Value
IRA	18.17 (3.43)	9.877 (1.29)	9.183 (0.48)	7.185 (0.50)	3.084 (0.18)	*p* < 0.0001
IOD	0.7442 (0.02)	0.6397 (0.02)	0.5605 (0.04)	0.5153 (0.02)	0.2452 (0.02)	*p* < 0.0001

Median values and standard deviations (SD) are shown for the percentage of DHH-immunoreactive area (%) and integrated optical density (IOD) in the interstitial Leydig cells of cats aged 6, 8, 12, 24, and 36 months (*n* = 5 per group). A significant reduction in both parameters was observed across age groups (*p* < 0.0001, Kruskal–Wallis test).

**Table 3:** Pearson correlation analysis between age and reproductive/body parameters in male subjects (*Felis silvestris catus*).

Comparison	Pearson’s r	R^2^	95% Confidence Interval	*p*-Value	Interpretation	Comparison
Age vs. BW	0.9302	0.8653	0.2674 to 0.9955	0.0219	Strong and significant positive correlation. Body weight increased significantly with age.	Age vs. BW
Age vs. TW	0.4344	0.1887	−0.7262 to 0.9519	0.4648	Weak correlation, not significant.	Age vs. TW
Age vs. Right T-index	0.3555	0.1264	−0.7675 to 0.9422	0.557	Weak correlation, not significant.	Age vs. Right T-index
Age vs. Left T-index	0.402	0.1616	−0.7442 to 0.9480	0.5023	Weak correlation, not significant.	Age vs. Left T-index
Age vs. GSI	0.8453	0.7146	−0.1453 to 0.9896	0.0713	Strong correlation, but not significant at 5%. May be biologically relevant.	Age vs. GSI

This table summarizes the Pearson correlation coefficients (r), coefficients of determination (R^2^), 95% confidence intervals, and *p*-values for the associations between age and various physiological parameters: body weight (BW), testicular weight (TW), right and left testicular index (T-index), and gonadosomatic index (GSI). A strong and statistically significant positive correlation was observed only for body weight. Other parameters showed weak or non-significant associations, although the correlation with GSI may have biological relevance despite not reaching conventional statistical significance (*p* < 0.05).

## Data Availability

All supplementary stereological, morphometric, and multivariate data supporting the findings of this study are openly available in https://doi.org/10.5281/zenodo.17954344.

## Supplementary Materials

The following supporting information can be downloaded at: https://www.mdpi.com/article/10.3390/ani16010010/s1, File S1, comprising graphical representations of age-dependent stereological dynamics (Figure S1); File S2, containing the complete numerical estimators for all stereological parameters (Table S1); and File S3, which provides the full numerical outputs of the Principal Component Analysis (PCA), including eigenvalues, percentage of explained variance, variable loadings for PC1 and PC2, and individual scores used to generate the PCA score and loading plots presented in Figure 3 (Table S2).

## Abbreviations

The following abbreviations are used in this manuscript:

AR	androgen receptor
BSA	Bovine Serum Albumin
BW	Body Weight
CE	Coefficient of Error
CV	Coefficient of Variation
DAB	3,3′-Diaminobenzidine
DHH	Desert Hedgehog
DNA	Deoxyribonucleic Acid
FIJI	Fiji ImageJ (Image Processing Software)
GSI	Gonadosomatic Index
HRP	Horseradish Peroxidase
ICVGAN	International Committee on Veterinary Gross Anatomical Nomenclature
ICVHN	International Committee on Veterinary Histological Nomenclature
IGF1	*Insulin-Like Growth Factor 1*
IOD	Integrated Optical Density
IQR	Interquartile Range
IUR	Isotropic Uniform Random (sectioning)
LH	Luteinizing Hormone
PBS	Phosphate-Buffered Saline
PCA	Principal Component Analysis
Q_A_	Numerical Density (cells/mm^2^)
SD	Standard Deviation
SEM	Standard Error of the Mean
SURS	Systematic Uniform Random Sampling
S_V_	Surface Density
TW	Testicular Weight
V_ABS_	Absolute Volume (mm^3^/testis)
Vol_REF_	Reference Volume
VV	Volume Density (Volume Fraction, %)
