# Supplementary material for: Quantitative Postnatal Maturation of the Feline Testis from 6 to 36 Months: A Stereological and DHH Immunomorphological Analysis
Source: Animals (Basel). 2025 Dec 19;16(1):10. doi: 10.3390/ani16010010 (PMC12785084; doi:10.3390/ani16010010)
Supplement: Supplementary file 1 [file animals-16-00010-s001.zip › File S1.pdf]

File S1

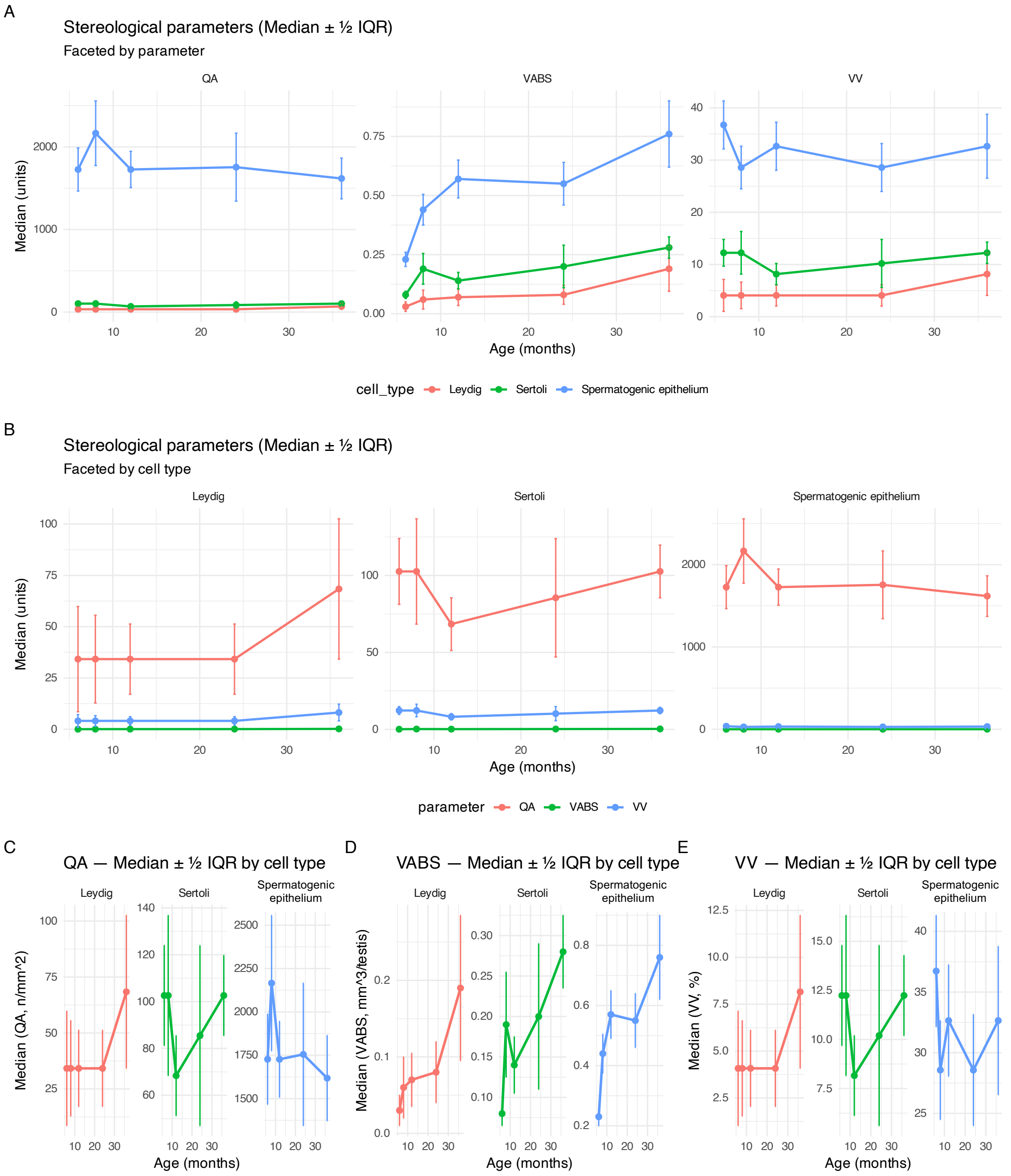

Figure S1. Age-dependent stereological dynamics of testicular compartments in domestic cats (*Felis silvestris catus*). (A) Global stereological estimators (median ± ½ IQR) for numerical density (Q_A), absolute volume (V_{ABS}), and volume fraction (V_V), shown across the three main cell populations: Leydig cells (red), Sertoli cells (green), and spermatogenic epithelium (blue). A progressive increase in V_{ABS} and V_V is evident with advancing age, particularly in the spermatogenic epithelium, while Q_A remained relatively stable. (B) Age-related distribution of stereological estimators separated by cell type. Leydig cells showed stable Q_A but a marked increase in V_{ABS}, consistent with cellular hypertrophy. Sertoli cells displayed volumetric expansion with moderate variation in Q_A. The spermatogenic epithelium exhibited the highest Q_A values, with modest fluctuations across ages. (C) Median Q_A values (cells/mm²) by cell type. The spermatogenic epithelium consistently showed the highest cell density, whereas Leydig and Sertoli cells maintained lower and more stable densities. (D) Median V_{ABS} (mm³/testis) by cell type. Both Sertoli cells and the spermatogenic epithelium increased in absolute volume with age, while Leydig cells displayed a significant volumetric expansion despite stable numerical density. (E) Median V_V (%) by cell type. The relative proportion of Sertoli cells and spermatogenic epithelium remained consistently higher than that of Leydig cells, with subtle fluctuations across developmental stages. All data are presented as median ± ½ interquartile range (IQR), obtained from cats aged 6, 8, 12, 24, and 36 months (n = 5 per group). Statistical comparisons were performed using the Kruskal–Wallis test with Dunn's post hoc analysis ($p < 0.05$ considered significant).
