# Supplementary material for: Quantitative Postnatal Maturation of the Feline Testis from 6 to 36 Months: A Stereological and DHH Immunomorphological Analysis
Source: Animals (Basel). 2025 Dec 19;16(1):10. doi: 10.3390/ani16010010 (PMC12785084; doi:10.3390/ani16010010)
Supplement: Supplementary file 1 [file animals-16-00010-s001.zip › File S2.pdf]

File S2

Table S1. Absolute and estimated stereological parameters in the testis of the domestic cat (*Felis silvestris catus*) at different ages. Median values and interquartile ranges (IQR) are presented for stereological estimators in the testis of cats aged 6, 8, 12, 24, and 36 months (n = 5 per group). Parameters include reference volume (Vol_{REF}, mm³), numerical density (Q_A, cells/mm²), absolute volume (V_{ABS}, mm³/testis), and volume fraction (V_V, %) of Leydig cells, Sertoli cells, and the spermatogenic epithelium. Estimates of surface density (SV) of seminiferous tubules and whole testis volume were also included. Observed coefficient of group variation ($\%OCV_{GROUP}$) and individual estimator error ($\%OCE_{INDIVIDUAL}$) are provided for each variable. Superscript numbers denote statistically significant differences between age groups ($p < 0.05$, Kruskal–Wallis test with Dunn's post hoc).

	Estimators		6m	8m	12m	24m	36m	p-value
Testis	Vol_{REF} (mm³)	MEDIAN (IQR)	0,34 (0,35)	1,45 (0,47)	1,56 (0,58)	1,75 (0,63)	2,15 (1,37)	< 0.0001
		$\%OCV_{GROUP}$	58,89	16,36	24,11	18,24	34,77	
		$\%OCE_{INDIVIDUAL}$	18,62	6,68	7,62	6,08	14,19	
Leydig cell	Q_A (nº/mm⁻²)	MEDIAN (IQR)	34,19 (51,29)	34,19 (42,73)	34,19 (34,19)	34,19 (34,19)	68,38 (68,38)	0.4538
		$\%OCV_{GROUP}$	83,76	83,73	70,14	70,77	61,94	
		$\%OCE_{INDIVIDUAL}$	16,75	16,74	14,02	14,15	12,38	
	V_{ABS} (mm³/testis)	MEDIAN (IQR)	0,03 (0,04)[1, 2]	0,06 (0,08)[3]	0,07 (0,07)	0,08 (0,08)[1]	0,19 (0,19)[2, 3]	< 0.0001
		$\%OCV_{GROUP}$	85.47	85.90	68.11	70.76	61.79	
		$\%OCE_{INDIVIDUAL}$	17,09	17,17	13,62	14,15	12,36	
	V_V (%)	MEDIAN (IQR)	4,08 (6,12)	4,08 (5,10)	4,08 (4,08)	4,08 (4,08)	8,16 (8,16)	0.4374
		$\%OCV_{GROUP}$	83.78	83.72	70.16	70.78	61.94	
		$\%OCE_{INDIVIDUAL}$	16,75	16,74	14,03	14,15	12,39	
Sertoli cell	Q_A (nº/mm⁻²)	MEDIAN (IQR)	102,6 (42,77)[1]	102,6 (68,42)	68,38 (34,19)[1]	85,47 (76,91)	102,6 (34,23)	0.0147
		$\%OCV_{GROUP}$	37.81	42.77	48.19	53.25	38.76	
		$\%OCE_{INDIVIDUAL}$	7,564	8,554	9,638	10,650	7,752	
	V_{ABS} (mm³/testis)	MEDIAN (IQR)	0,08 (0,03)[1,2,3,4]	0,19 (0,13)[1,5]	0,14 (0,07)[2,6]	0,20 (0,18)[3]	0,28 (0,09)[4,5,6]	< 0.0001
		$\%OCV_{GROUP}$	37.04	43.19	47.10	53.25	39.09	
		$\%OCE_{INDIVIDUAL}$	7,41	8,64	9,41	10,65	7,81	
	V_V (%)	MEDIAN (IQR)	12,24 (5,11)	12,24 (8,17)	8,16 (4,08)	10,20 (9,19)	12,24 (4,09)	0.0147
		$\%OCV_{GROUP}$	37.83	42.79	48.2	53,27	38.78	
		$\%OCE_{INDIVIDUAL}$	7,56	8,55	9,64	10,65	7,75	
Spermatogenic epithelium	Q_A (nºcell/mm⁻²)	MEDIAN (IQR)	1727 (521)[1]	2166 (782)[1,2,3,4]	1727 (439)[2]	1755 (823)[3]	1618 (493)[4]	0.0353
		$\%OCV_{GROUP}$	20,31	28,36	25,19	32,93	17,58	
		$\%OCE_{INDIVIDUAL}$	4,06	5,67	5,03	6,58	3,51	

	V_{ABS} (mm^3/testis)	MEDIAN (IQR)	0,23 (0,06)^{1,2,3,4}	0,44 (0,13)^{1,5}	0,57 (0,16)^{2}	0,55 (0,18)^{3}	0,76 (0,28)4,5	< 0.0001
		%OCV$_{GROUP}$	15,66	22.33	20.07	27.63	25.12	
		%OCE$_{INDIVIDUAL}$	3,13	4,46	4,01	5,52	5,02	
	V_V (%)	MEDIAN (IQR)	36,73 (9,19)^{1,2,3,4}	28,57 (8,16)^{1}	32,65 (9,19)^{2}	28,57 (9,18)^{3}	32,65 (12,24)^{4}	< 0.0001
		%OCV$_{GROUP}$	15.05	22.21	20.07	27.70	25.09	
		%OCE$_{INDIVIDUAL}$	3,011	4,442	4,013	5,540	5,019	

Median values and interquartile ranges (IQR) are presented for stereological estimators in the testis of cats aged 6, 8, 12, 24, and 36 months (n = 5 per group). Parameters include reference volume (VolREF, mm^3), numerical density (QA, cells/mm^2), absolute volume (VABS, mm^3/testis), and volume fraction (VV, %) of Leydig cells, Sertoli cells, and the spermatogenic epithelium. Estimates of surface density (SV) of seminiferous tubules and whole testis volume were also included. Observed coefficient of group variation (%OCVGROUP) and individual estimator error (%OCEINDIVIDUAL) are provided for each variable. Superscript numbers denote statistically significant differences between age groups ($p < 0.05$, Kruskal–Wallis test with Dunn's post hoc).
