# Supplementary material for: Quantitative Postnatal Maturation of the Feline Testis from 6 to 36 Months: A Stereological and DHH Immunomorphological Analysis
Source: Animals (Basel). 2025 Dec 19;16(1):10. doi: 10.3390/ani16010010 (PMC12785084; doi:10.3390/ani16010010)
Supplement: Supplementary file 1 [file animals-16-00010-s001.zip › File S3.pdf]

File S3

Table S2. Standardized principal component loadings for stereological and morphometric variables of the feline testis

Var	PC1	PC2	PC3
AV	0,984700007005213	-0,00189141311290397	-0,159934435099976
VV[germ epith]	-0,830881222098754	0,465553183785556	-0,304702975356216
V[germ epith]	0,943581705173206	0,0891584275740452	-0,288527977123844
VV[inters_space]	0,796820634614695	-0,278856741815597	0,535982726558414
V[inters_space]	0,994493781370535	-0,0142230723288269	0,103825095483024
VV[LumenST]	0,394449997243275	-0,741028267414082	-0,296059801517012
V[LumenST]	0,961007882354069	-0,15769128028491	-0,219186264293846
SV[LumenST]	-0,340828042986536	-0,697440370648983	0,630337638914872
Surf[LumenST]	0,983691337615145	-0,151198005963744	-0,0724006305682148
VV[Leydig cell]	0,320904505157331	0,915040250859558	0,0608072443820263
V[Leydig cell]	0,945343005319689	0,311686255239004	-0,0957521589773226
VV[Sertoli cell]	-0,158607949579311	0,745396291048317	0,628287357827768
V[Sertoli cell]	0,947330899023131	0,300791787917505	0,103122190750799
QA[Leydig cell]	0,342096121702441	0,54085272911086	0,768091004996848
QA(Germ cell]	0,0831568785274922	-0,532426076742366	0,840720553968795

This table presents the standardized loadings of all stereological and morphometric variables included in the principal component analysis (PCA) across the first three principal components (PC1, PC2, and PC3). Loadings represent the direction and magnitude of the contribution of each variable to the corresponding component. PC1 primarily reflects global testicular growth and volumetric expansion of the main compartments, PC2 is mainly associated with variation in volume fractions and cellular densities, and PC3 captures residual variance related to surface-related parameters and germ cell density. Variables with higher absolute loading values exert a stronger influence on component definition.

Abbreviations: AV, absolute testicular volume; VV, volume fraction; V, absolute volume; QA, numerical density (cells/mm^2); SV, surface density; Surf, surface area; ST, seminiferous tubules; germ epith, spermatogenic epithelium; inters space, interstitial space.
